# Assessment and Correlates of Different Phenotypical Characteristics of Psychological Flexibility in Adapting to Chronic Pain: A Feasibility Study

**DOI:** 10.1111/papr.70047

**Published:** 2025-05-22

**Authors:** Ivo P. Legierse, Henriët van Middendorp, Maike Borgonjen, Ewald M. Bronkhorst, Martijn F. Pisters, Kris C. P. Vissers, Monique A. H. Steegers

**Affiliations:** ^1^ Department of Anaesthesiology, Pain and Palliative Medicine Radboud University Medical Centre Nijmegen the Netherlands; ^2^ Health, Medical and Neuropsychology Unit, Faculty of Social and Behavioural Sciences, Institute of Psychology Leiden University Leiden the Netherlands; ^3^ Pro Persona Mental Healthcare Nijmegen the Netherlands; ^4^ Department for Health Evidence Radboud University Medical Center Nijmegen the Netherlands; ^5^ Research Group Empowering Healthy Behaviour, Department of Health Innovation and Technology Fontys University of Applied Sciences Eindhoven the Netherlands; ^6^ Physical Therapy Research, Department of Rehabilitation, Physical Therapy Science and Sport, Brain Center Rudolf Magnus University Medical Center Utrecht Utrecht the Netherlands; ^7^ Department of Anesthesiology University Medical Center Amsterdam Amsterdam the Netherlands

**Keywords:** adaptation, biopsychosocial model, chronic pain, health, phenotypic variability, psychological flexibility

## Abstract

**Objectives:**

This study examined the feasibility of a research protocol for assessing psychological flexibility in patients with chronic pain to gain insight into the uniqueness of different phenotypes of psychological flexibility and to tentatively test whether psychological flexibility is associated with effective adaptation to chronic pain.

**Methods:**

In a cross‐sectional study, in twenty patients with chronic pain, different phenotypes of psychological flexibility and a variety of positive and negative health indicators were assessed. Correlations were explored to determine the unicity of the different phenotypes of psychological flexibility and to test their associations with chronic pain.

**Results:**

All phenotypes of psychological flexibility could be assessed reliably in this patient group. Preliminary findings suggest that all phenotypes assess unique flexibility aspects (79% of the intercorrelations were less than moderate; > −0.30, < 0.30). Higher levels of different psychological flexibility phenotypes were generally associated with higher positive health indicators and lower negative health indicators (70% of the moderate correlations; ≤ −0.30 or ≥ 0.30 were in the expected direction).

**Conclusions:**

Results confirm that the protocol is feasible for large‐scale research in patients with chronic pain and that it is useful to further investigate the different phenotypes of psychological flexibility in relation to optimal adaptation to chronic pain in a longitudinal study.

**Practice Implications:**

Psychological flexibility is a potentially important future target in the treatment (e.g., biofeedback, cognitive behavioral therapy, mindfulness) of patients with chronic pain.

## 1 Introduction

Chronic pain is one of the most prevalent health problems in the world [1], being defined as an unpleasant sensory and emotional experience associated with, or resembling that associated with, actual or potential tissue damage [2]. Overall, nearly 1 in 5 citizens will experience chronic pain during their lifetime [3]. Chronic pain is multidimensional in nature [4], demonstrating the influence of biological, social, existential, and psychological factors in the development, increase, or maintenance of chronic pain [5, 6, 7].

Chronic pain provides a substantial burden on the patients' social environment, employers, healthcare systems, and society in general [8]. For the individual patient, chronic pain impacts quality of life (QoL) and participation in, for example, family life, social activities, and work [3, 9, 10].

Despite a general negative impact, there is no direct correlation between the level of pain that patients experience and their QoL and level of participation [11, 12]. Since each patient deals with chronic pain differently, there is variation in adaptation. This implies that individual characteristics might play a meaningful role in adaptation to the impact of chronic pain for individual patients.

A lot of research has been done on psychological factors that impact the development and maintenance of chronic pain, such as negative affect, distress, trauma, and catastrophizing [13]. Yet, much less is known about psychological factors that are associated with adaptation to chronic pain, which could contribute to experiencing a life that is as healthy and meaningful as possible despite the pain. This should be clarified in order to develop new, more personalized multidimensional treatment strategies for patients, or it may contribute to the prevention of the development or maintenance of chronic pain.

Previous reviews have shed some first light on the psychological adaptation to chronic diseases and refer to the fact that it is a dynamic, continuous process, facilitating a balance in different domains of life, and an experience of optimal QoL, in which physiological, cognitive, emotional, and behavioral aspects and goal adjustment play a role [14, 15, 16, 17]. It is largely unclear which individual factors contribute to optimal adaptation; however, a promising psychological factor in the face of adaptation is psychological flexibility. Kashdan and Rottenberg have demonstrated that psychological flexibility plays an important role in (psychological) health in relation to the continuously changing nature of everyday life [18]. The core of psychological flexibility refers to the ability to continually respond and adapt to changing circumstances and experiences, such as thoughts, feelings, and events, over time, thereby balancing competing desires, needs, and life domains, and enhancing optimal well‐being [18].

Because of the multidimensional nature of the concept of psychological flexibility, different phenotypes have been distinguished. *Cognitive flexibility* is the ability to shift a course of thought or action according to the changing demands of the situation [19]. *Emotional* or *expressive flexibility* is the ability to both enhance and suppress emotion in the face of situational demands [20]. *Behavioral* or *coping flexibility* is the ability to modify one's coping strategies adaptively to meet the demands of different stressful situations [21]. *Goal setting flexibility* or *goal adjustment capacities* have been defined as individual differences in goal disengagement and goal reengagement capacities when a goal becomes unattainable [22]. Finally, *psychophysiological flexibility* is seen as the ability of the parasympathetic nervous system to return to a resting state or homeostasis in the face of constantly changing physiological and environmental demands. Heart Rate Variability (HRV) is a relevant biomarker of this capacity to adapt physiologically [23, 24].

**FIGURE 1:**
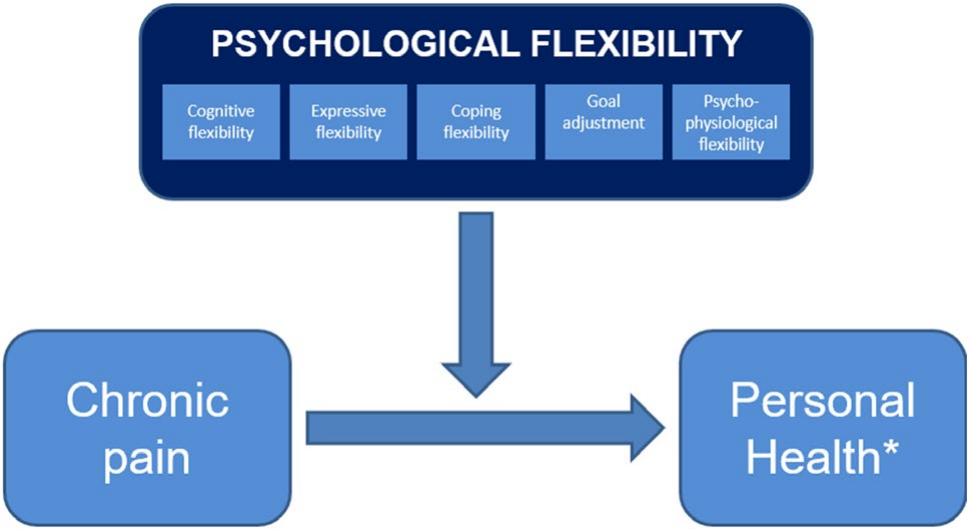
Conceptual model on the role of psychological flexibility in the adaptation to chronic pain. *Personal health is operationalized using various positive and negative health indicators. In this study: Mental well‐being, participation, pain, fatigue, anxiety, depression, pain catastrophizing, and health care consumption.

For patients suffering from chronic pain, all phenotypes of flexibility appear to be of added value in relation to adequate adaptation, as they are associated with a diversity of physical and mental health indicators in different chronic pain samples [25, 26, 27, 28, 29, 30, 31, 32, 33, 34]. To the best of our knowledge, all different phenotypes of psychological flexibility have never been investigated simultaneously (Figure 1). It is therefore still largely unclear whether there is conceptual overlap and whether, and to what extent, they make a unique contribution to adaptation to chronic pain. To gain more insight into this, it is therefore necessary to study all phenotypes simultaneously.

The primary aim of this study is to assess the feasibility of the measurement of aforementioned different phenotypes of psychological flexibility in a heterogeneous chronic pain population. In addition to feasibility, a tentative exploration will be conducted of whether the different phenotypes of flexibility described in the literature are unique or whether there is an overarching flexibility construct, and the relationship between these phenotypes of flexibility and positive and negative health indicators to gain insight into the ability of patients to adapt to chronic pain.

## 2 Methods

### 2.1 Participants

Participants for this feasibility study were recruited from an academic ambulatory pain center, who were referred by their general practitioner. To participate in this study, patients had to meet all the following inclusion criteria: chronic pain (≥ 3 months), age between 18 and 70 years proficiency in the Dutch language, and provide written consent. Exclusion criteria were: sedating medication that influenced daily cognitive functioning at the time of the study, current severe psychiatric disorder (e.g., schizophrenia, severe behavioral disorder, major depressive episode, panic or anxiety disorder) interfering with at least moderate to normal functioning at the time of the study, major vision or hearing problem or major motoric impairment causing problems executing neuropsychological tests, and atrioventricular (AV) conduction disorder for which the patient receives medication, because it affects the beat‐to‐beat intervals, which is important for measuring HRV.

### 2.2 Procedure and Design

In a cross‐sectional design, a member of the treatment team contacted all patients who met the inclusion criteria by telephone prior to their appointment at the pain center, providing general information about the study. If patients showed interest in the study, they received written information. After three to 7 days, patients were called to assess willingness to participate. During their appointment at the pain center, a physician decided whether a patient could participate definitively in the study based on the exclusion criteria. Written informed consent was obtained from the patient. Table 1 provides an overview of all measurements. Participants completed part of the questionnaires online prior to their appointment, as a part of the routine measurements of the pain center; the remaining questionnaires were administered directly after the appointment with the physician at the pain center. The questionnaires for this study were completed by participants on a computer, which took approximately 25 min. Besides these aforementioned measures, several demographic and disease‐related variables were assessed (Table 2). After completing the questionnaires, two neuropsychological tests were administered by the researcher, which took approximately 20 min, and a 3‐min HRV measurement. HRV measurements were on the short side and we did not consider the influence of confounding factors (e.g., alcohol, caffeine) [35].

**TABLE 1:** Overview measures.

	Construct	Instrument and publication(s)	Scales (internal consistencies of publication | this sample) and example item	Interpretation of score(s)
Flexibility measures	Cognitive flexibility—Task switching	Letter‐Number Test taken with INQUISIT Millisecond (v.5.0.14.0, 2018) WA, Seattle [36, 37]	Switch cost is measured in milliseconds by the difference between the mean correct latency of switch trials and mean of non‐switch trials. A positive value of switch cost means participants were slower on switch trials Internal consistency is not applicable	Smaller switch costs indicate a higher level of cognitive flexibility^c^
	Cognitive flexibility—inhibition	Stop‐signal test taken with INQUISIT Millisecond (v.5.0.14.0, 2018) WA, Seattle [38, 39]	Stop Signal Reaction Time (SSRT) is the time in milliseconds required to stop the initiated go process, calculated by the difference between the mean reaction time for all no‐signal trials and the mean of stop‐signal delays Internal consistency is not applicable	A shorter SSRT indicates less difficulty inhibiting a response^c^
	Expressive flexibility	Flexible Regulation of Emotional Expression (FREE) scale^a^ [40, 41]	*Enhancement of emotions* (α = 0.81|*α* = 0.83); “A friend wins an award for a sport that doesn't interest you” *Suppression of emotions* (α = 0.70|*α* = 0.65); “After you have a very irritating and stressful day, a sometimes‐annoying neighbor stops by to say hello” *Expressive Flexibility (EF)*: ability to flexibly enhance and suppress emotional Expression in accordance with different contexts. EF is calculated by subtracting the absolute difference between the scores of enhancement and suppression from the sum of the scores on enhancement and suppression	Higher scores of EF indicate greater flexibility in regulating expression of emotions
	Coping flexibility	Coping Flexibility Questionnaire (COFLEX) [42]	*Versatility* (*α* = 0.94|*α* = 0.88): capability of a person to flexibly use different coping strategies in accordance with personal goals and situational limitations; “I immediately change my approach if a certain approach fails” *Reflective coping* (*α* = 0.72|*α* = 0.70): ability to generate, consider, and assess the suitability of different coping alternatives in a given situation; “I question myself whether my approach to the problem is the best solution”	Higher scores mean better coping flexibility abilities

	Goal adjustment	Goal Adjustment Scale (GAS) [22]	*Goal Disengagement* (α = 0.77|*α* = 0.84): capacity to withdraw from the pursuit of an unattainable goal; “It's easy for me to reduce my effort towards the goal” *Goal Reengagement* (*α* = 0.83|*α* = 0.86): capacity to identify, commit, and pursue new goals when unattainable goals exist; “I seek other meaningful goals”	Higher scores mean a better capacity to adjust personal goals
Psycho‐ physiological flexibility	Heart rate variability (HRV) [35, 43, 44, 45]	HRV is collected using a Polar H7 belt (Polar Electro, Kempele, Finland) and EliteHRV application conform the recommendations of Laborde and colleagues. HRV was assessed by computing the root mean square of successive differences (RMSSD), which is the preferred measurement for short‐term assessment of (parasympathetic) activity in the autonomous nervous system. Due to the total time of the study protocol, we did a three‐minute measurement instead of the often recommended 5 min. The collected beat‐to‐beat intervals were imported into Kubios HRV 3.4 for further analysis. Using the Kubios medium threshold‐based beat correction level algorithm, which identifies inter‐beat‐intervals that are larger or smaller than 0.25 s compared with the average, artifacts were filtered out	Higher HRV scores are considered a measure of a higher degree of psychophysiological flexibility
Health indicators	Mental well‐being	Mental Health Continuum‐Short Form (MHC‐SF) [46, 47, 48]	*Emotional well‐being*: feelings of happiness, satisfaction, and interest in life; “How often did you feel happy?” *Psychological well‐being*: the subjective evaluation of optimal individual functioning; “How often did you feel that you liked most parts of your personality?” *Social well‐being*: the subjective evaluation of optimal functioning for a community “How often did you feel that you had something important to contribute to society?” *Mental well‐being*: a combination of emotional, psychological and social well‐being (α = 0.93 | α = 0.89) Mental well‐being is the total score of the 3 subscales	Higher scores indicate greater levels of mental well‐being

	Participation and Autonomy	Impact on Participation and Autonomy Questionnaire (IPA) [49]	*Autonomy indoors* (*α* = 0.96|*α* = 0.91): “My chances of getting around in my house where I want to are…” *Family Role* (*α* = 0.93|*α* = 0.90): “My chances of getting housework done, either by myself or by others, when I want them done are…” *Autonomy Outdoors* (*α* = 0.92|*α* = 0.81): “My chances of seeing people as often as I want are…” *Social Relations* (*α* = 0.86|*α* = 0.86): “My relationships with acquaintances are…” *Work and education* (*α* = 0.85|*α* = 0.91): “My chances of getting different paid or voluntary work are…”	Higher scores indicate that a person experiences *more* barriers to participation and autonomy. There is no total score of participation^c^
	Pain	Visual Analogue Scale (VAS)	Current pain on a 100‐point scale varying from 0 “no pain” to 100 “worst pain possible” Internal consistency is not applicable	Higher scores indicate worse pain
	Fatigue	Visual Analogue Scale (VAS)	Current fatigue on a 100‐point scale varying from 0 “no fatigue” to 100 “worst fatigue possible” Internal consistency is not applicable	Higher scores indicate worse fatigue
	Anxiety and depression^b^	Hospital Anxiety and Depression Scale (HADS) [50, 51]	*Anxiety* (*α* = 0.84|0.83): “I feel tense or wound up” *Depression* (*α* = 0.84|0.87): “I have lost interest in my appearance”	Higher scores indicate more feelings of Anxiety or Depression
	Pain Catastrophizing^b^	Pain Catastrophizing Scale (PCS) [52, 53]	*Rumination*: “I worry all the time about whether the pain will end” *Magnification*: “It's terrible and I think it's never going to get any better” *Helplessness*: “There's nothing I can do to reduce the intensity of the pain” *Pain catastrophizing*: an exaggerated negative mental set brought to bear during actual or anticipated painful experience. (*α* = 0.87|*α* = 0.92) is the total score of the three subscales	Higher scores indicate a higher level of catastrophizing.
Healthcare consumption	Not applicable	A 6‐point ordinal scale indicating self‐reported number of doctor's visits last 12 months, ranging from “none” to “more than 20 times”	Higher scores indicate more healthcare consumption

^a^ FREE was translated in Dutch and then back‐translated into English by officially certified translators. The back‐translated English version was read by one of the authors of the original FREE scale, who indicated that the back translation matches the original questionnaire.

^b^ Questionnaires completed online prior to the physician's appointment.

^c^ For interpretation matters, the polarity of the score on this measure has been reversed during statistical analysis.

**TABLE 2:** Sample characteristics.

Characteristic	*N* (%)	*M* ± SD	Range
Age	20 (100%)	52.8 ± 11.9	28–67
Sex			
Male	8 (40%)		
Female	12 (60%)		
BMI		26.5 ± 5.4	16.9–39.7
Highest educational level			
Primary education	0 (0%)		
Secondary education	15 (75%)		
Tertiary education	5 (25%)		
Marital status			
Living with partner	15 (75%)		
Living without partner	5 (25%)		
Employed			
Yes	9 (45%)		
No	11 (55%)		
Duration of pain complaints			
3–6 months	0 (0%)		
6–12 months	4 (20%)		
1–2 year	1 (5%)		
Longer than 2 years	15 (75%)		
Localisation of pain^a^			
Lower extremities	12 (60%)		
Back	10 (50%)		
Abdomen	4 (20%)		
Upper extremities + shoulders	4 (20%)		
Head + neck	2 (10%)		
Thorax	0 (0%)		

^a^ Patients could indicate multiple locations.

The study (protocol number 2018–4868) has been reviewed and approved by the ethics committee of the Radboud University Nijmegen Medical Center.

### 2.3 Measures

A diversity of psychological flexibility measures and positive and negative health indicators were completed by all participants. Information on the measurement instruments used, their (sub)scales, internal consistency, and interpretation can be found in Table 1.

### 2.4 Statistical Analysis

To examine the associations between the different phenotypes of psychological flexibility and between the different flexibilities and positive and negative health indicators, Pearson correlations were calculated, except for Health care consumption, for which Spearman correlations were calculated due to the skewed distribution and nature of the answer categories. Correlation coefficients with absolute values between 0.10 and 0.30 were considered weak, between 0.30 and 0.50 as moderate, and ≥ 0.50 as strong [54]. All statistical tests were two‐tailed and significant results (*p* < 0.05) are noted as such, but due to the small sample size, significance is considered less important than the size of the correlations. Statistical analyses were conducted using SPSS, version 29.0 (IBM SPSS Statistics, Somers, N.Y.). Due to reversing polarity of three measurements (Table 1), higher scores on all flexibility measures indicate more flexibility, higher scores on all positive health indicators indicate a better health outcome, and higher scores on all negative health indicators indicate a worse health outcome.

## 3 Results

### 3.1 Participant Characteristics

In total, 21 patients participated in the study. One patient dropped out during the study due to excessive pain, which was not induced or aggravated by performing the research protocol. Data of this dropout has not been included in this study. The mean age of the participants was 52.8 years, and 60% were female and 40% male. The participants were heterogeneous in the localization of their pain (Table 2). The most commonly reported localization of pain was the lower extremities (60%) and the back (50%) and, as expected in an academic pain center, in the majority of participants (75%) the pain symptoms were long‐lasting (longer than 2 years), and the mean pain level was high (*M* = 73.75, SD = 13.46, range 50–100). Mean scores on depression and anxiety were below the cut‐off score of 8, indicating no mental distress. However, further analysis indicated that 45% of the participants (*N* = 9) scoring ≥ 8 on depression, and 45% (*N* = 9) scoring ≥ 8 on anxiety, indicating a possible depression or anxiety disorder. The mean score on pain catastrophizing was also high (*M* = 24.30, SD = 10.80, range 9–52), but in accordance with expectations in a chronic pain population. A cut‐off of more than 30 points [52] has been shown to be associated with clinical relevance, which was the case for six participants.

### 3.2 Descriptive Statistics

Descriptive analyses were conducted on the eight different flexibility measures (Table 3). The two measures of cognitive flexibility show quite a lot of variation in reaction time in both the switching task (*M* = 612 ms, SD = 310 ms) and the inhibition task (*M* = 252 ms, SD = 62 ms). Since four participants did not meet the cut‐off score for participation in the task‐switching test, the results are based on sixteen participants. Results on the self‐reported flexibility measures showed generally moderate scores with relatively limited variation: Expressive Flexibility (*M* = 3.69, SD = 0.69), Coping Flexibility *Versatility* (*M* = 2.65, SD = 0.72), Coping Flexibility *Reflective* (*M* = 2.54, SD = 0.66), Goal Disengagement (*M* = 2.56, SD = 0.70), and Goal Reengagement (*M* = 3.18, SD = 0.62). Results on the psychophysiological flexibility measure (HRV) showed a lot of variation (*M* = 25, SD = 15). All HRV measurements were 3 min, except for one. Due to a large amount of noise, the first 30 s were deleted in this measurement.

**TABLE 3:** Characteristics of instruments.

	Construct	Instrument/scales	*N* (%)	*M* ± SD	Range
Flexibility	*Cognitive – switching*	Letter‐number task (ms)	16 (80%)	612.39 ± 309.56	−16.04—1059.23
	*Cognitive—inhibition*	Stop signal Test (ms)	20 (100%)	251.68 ± 62.31	78.33–370.95
	*Expressive*	FREE expressive flexibility (16–96)	20 (100%)	59.00 ± 11.11	40–80
	*Coping*	COFLEX versatility (0–4)	20 (100%)	2.65 ± 0.72	1.44–4.00
		COFLEX reflective coping (0–4)	20 (100%)	2.54 ± 0.66	1.50–4.00
	*Goal adjustment*	GAS goal disengagement (1–5)	20 (100%)	2.56 ± 0.70	1.50–3.75
		GAS goal reengagement (1–5)	20 (100%)	3.18 ± 0.62	1.83–4.00
	*Psychophysiological—HRV*	RMSSD	20 (100%)	25.13 ± 15.43	9.00–68.10
Positive and negative health indicators	*Mental Well‐being*	MHCS‐F—total score (0–5)	20 (100%)	2.82 ± 1.21	0.71–4.21
	*Participation and Autonomy*	IPA autonomy indoors (0–4)	20 (100%)	1.16 ± 1.03	0.00–3.43
		IPA family role (0–4)	20 (100%)	2.03 ± 1.07	0.00–3.86
		IPA autonomy outdoors (0–4)	20 (100%)	1.96 ± 1.09	0.00–4.00
		IPA social relations (0–4)	20 (100%)	1.35 ± 0.82	0.00–3.57
		IPA work and education (1–5)	13 (65%)	2.42 ± 1.08	1.00–4.00
	*Current pain level*	VAS pain (0–100)	20 (100%)	73.75 ± 13.46	50–100
	*Current fatigue level*	VAS fatigue (0–100)	20 (100%)	58.75 ± 34.75	0–95
	*Anxiety*	HADS‐anxiety (0–21)	20 (100%)	6.90 ± 4.44	0–16
	*Depression*	HADS‐depression (0–21)	20 (100%)	7.35 ± 5.09	0–18
	*Pain catastrophizing*	PCS—total score	20 (100%)	24.30 ± 10.80	9–52
	*Healthcare Consumption*	None	0 (0%)		
	*(Indicated by doctor's visits last 12 months)*	*1–2 times*	0 (0%)		
		*3–5 times*	1 (5%)		
		*6–10 times*	8 (40%)		
		*11–20 times*	3 (15%)		
		*More than 20 times*	8 (40%)		

Abbreviation: ms, milliseconds.

### 3.3 Feasibility

Completing the entire study protocol took participants approximately 45–60 min and proved easy to complete for all participants. The research protocol was easily applicable. The instructions for the neuropsychological tests were clear to all participants and ensured adequate administration. No questions were asked or ambiguities were reported when completing the questionnaires. Administration of the HRV measurement also presented no practical problems and no objections were expressed by the participants. The instruments provided valid measurements of the different psychological flexibilities and positive and negative health indicators, showing sufficient variability. No potential risks or practical issues have been identified that warrant modification of the protocol for future research.

### 3.4 Measures of Psychological Flexibility and Health in Patients With Chronic Pain

To get a first impression of the uniqueness of all flexibility measures, the correlations between the eight different flexibility measures were explored by means of Pearson correlations. Besides six moderate to (very) strong correlations (*r* = −0.38 to *r* = 0.71), 22 of the total of 28 correlations (79%) were less than moderate (Table 4).

**TABLE 4:** Correlations between the different psychological flexibility measures, including confidence intervals for the moderate to strong correlations.

Flexibility	1.	2.	3.	4.	5.	6.	7.
1. Cognitive—switching							
2. Cognitive—inhibition	0.004 (0.989)						
3. Expressive	0.044 (0.871)	−0.093 (0.695)					
4. Coping—versatility	−0.079 (0.770)	−0.120 (0.614)	0.223 (0.345)				
5. Coping—reflective	−0.214 (0.426)	0.139 (0.559)	0.160 (0.502)	**0.567** ** (0.009) [0.166, 0.807]			
6. Goal disengagement	−0.210 (0.436)	**−0.493** * (0.027) [−0.768, −0.064]	−0.015 (0.949)	−0.210 (0.373)	−0.170 (0.474)		
7. Goal reengagement	−0.181 (0.503)	**−0.376** (0.102) [−0.702, 0.080]	0.181 (0.446)	**0.505** * (0.023) [0.080, 0.774]	0.121 (0.610)	**0.467** * (0.038) [0.031, 0.754]	
8. Psychophysiological—HRV	−0.209 (0.437)	**0.714** **, * (<0.001) [0.398, 879]	−0.055 (0.817)	−0.144 (0.545)	0.213 (0.367)	−0.180 (0.447)	−0.061 (0.799)

*Note:* Ns for all flexibilities is 20, except for Cognitive flexibility—Switching (*N* = 16). Polarities are reversed for both Cognitive Flexibility measures: Switching and Inhibition, with higher scores indicating greater flexibility. The correlations highlighted in green indicate positive correlations ≥ 0.30, and the correlations highlighted in red indicate negative correlations ≤ −0.30. Exact *p*‐values are in parentheses (), and confidence intervals of the correlations (≤ −0.30 or ≥ 0.30) are in square brackets [].

* *p* < 0.05.

** *p* < 0.01.

*** *p* < 0.001.

To what extent the different psychological flexibility measures are potentially buffering the severity and influence of the pain on the patient's life, a series of correlation analyses with positive and negative health indicators are included in Table 5 and Table 6. In total, the flexibilities showed a moderate to strong relationship with 19 of the 48 (40%) positive health indicators (Table 5). Of these correlations, 74% (14/19) were in the expected direction, meaning that higher levels of flexibility are correlated with higher levels of positive health indicators, suggesting a more effective adaptation within a group of patients with chronic pain. In addition, the flexibility measures showed moderate to strong correlations with 11 of the 48 (23%) negative health indicators (Table 6), of which 64% (7/11) were in the expected direction; higher levels of flexibility were correlated with lower levels of negative health indicators.

**TABLE 5:** Correlations between the different phenotypes of psychological flexibility and positive health indicators, including confidence intervals for the moderate to strong correlations.

Flexibility	Correlations					
	Mental well‐being	Autonomy indoors	family role	Autonomy outdoors	Social relations	Work and eduction
Cognitive—switching	0.298 (*N* = 16) (0.263)	0.182 (*N* = 16) (0.500)	0.012 (*N* = 16) (0.964)	0.175 (*N* = 16) (0.517)	**0.353** (*N* = 16) (0.179) [−0.173, 0.722]	0.148 (*N* = 10) (0.684)
Cognitive—inhibition	0.130 (0.584)	−0.230 (0.330)	−0.126 (0.596)	**−0.422** (0.064) [−0.729, 0.025]	−0.268 (0.254)	0.198 (*N* = 13) (0.516)
Expressive	0.277 (0.236)	0.102 (0.668)	0.239 (0.311)	**0.330** (0.155) [−0.132, 0.674]	0.282 (0.228)	0.563* (*N* = 13) (0.045) [0.018, 0.850]
Coping—versatility	**0.318** (0.172) [−0.145, 0.667]	**0.385** (0.094) [−0.070, 0.707]	**0.439** (0.053) [−0.004, 0.738]	**0.381** (0.097) [−0.074, 0.705]	**0.348** (0.133) [−0.112, 0.685]	**0.318** (*N* = 13) (0.289) [−0.282, 0.740]
Coping—reflective	0.206 (0.382)	−0.026 (0.912)	−0.177 (0.457)	−0.160 (0.500)	−0.099 (0.677)	−0.135 (*N* = 13) (0.661)
Goal disengagement	−0.096 (0.687)	−0.153 (0.519)	−0.118 (0.619)	0.101 (0.672)	−0.114 (0.632)	**−0.403** (*N* = 13) (0.172) [−0.781, 0.190]
Goal reengagement	**0.306** (0.189) [−0.158, 0.659]	**0.346** (0.135) [−0.114, 0.684]	**0.433** (0.056) [−0.012, 0.735]	**0.603** ** (0.005) [0.218, 0.825]	**0.448** * (0.048) [0.006, 0.743]	−0.037 (*N* = 13) (0.904)
Psychophysiological—HRV	−0.028 (0.905)	**−0.467** * (0.038) [−0.754, −0.031]	**−0.374** (0.105) [−0.700, 0.083]	**−0.399** (0.081) [−0.716, 0.052]	−0.177 (0.457)	−0.146 (*N* = 13) (0.634)

*Note:* Ns for all flexibilities is 20, except for the correlations where a specific *N* is indicated in the table in parentheses () after the correlation coefficient. Polarities are reversed for both Cognitive Flexibility measures: Switching and Inhibition, with higher scores indicating greater flexibility. Besides these measures of flexibility, polarities are reversed for the Participation and Autonomy measures (Autonomy Indoors, Family Role, Autonomy Outdoors, Social Relations, Work and Education); higher scores mean less experience of barriers to participation and autonomy. The correlations highlighted in green indicate a positive correlation of ≥ 0.30 and the correlations highlighted in red indicate correlations ≤ −0.30. Exact *p*‐values are in parentheses (), and confidence intervals of the correlations (≤ −0.30 or ≥ 0.30) are in square brackets [].

* *p* < 0.05.

** *p* < 0.01.

**TABLE 6:** Correlations between the different phenotypes of psychological flexibility and negative health indicators, including confidence intervals for the moderate to strong correlations.

Flexibility	Correlations					
	Pain	Fatigue	Anxiety	Depression	Pain catastrophizing	Healthcare consumption
Cognitive—switching	0.064 (0.813)	−0.166 (0.539)	0.062 (0.819)	−0.155 (0.566)	−0.284 (0.287)	0.037 (0.891)
Cognitive—inhibition	0.084 (0.724)	0.019 (0.938)	−0.123 (0.605)	−0.278 (0.236)	**−0.576** ** (0.008) [−812, −180]	0.249 (0.290)
Expressive	−0.132 (0.579)	−0.050 (0.835)	−0.111 (0.641)	−0.183 (0.439)	**−0.323** (0.165) [−0.669, 0.140]	−0.238 (0.312)
Coping—versatility	**−0.340** (0.143) [−0.680, 0.121]	**−0.337** (0.146) [−0.679, 0.124]	−**0.337** (0.146) [−0.679, 0.124]	−0.290 (0.215)	**−0.351** (0.129) [−0.687, 0.108]	−0.282 (0.228)
Coping—reflective	−0.113 (0.636)	0.048 (0.841)	−0.246 (0.296)	−0.114 (0.633)	−0.226 (0.338)	−0.269 (0.252)
Goal disengagement	0.016 (0.947)	0.405 (0.076) [−0.045, 0.719]	0.023 (0.922)	0.231 (328)	**0.357** (0.123) [−0.102, 0.690]	**0.369** (0.109) [−0.101, 0.705]
Goal reengagement	−0.093 (0.698)	0.013 (0.958)	**−0.310** (0.184) [−0.662, 0.154]	−0.224 (0.342)	−0.013 (0.955)	0.062 (0.795)
Psychophysiological—HRV	0.285 (0.223)	0.071 (0.767)	−0.113 (0.634)	−0.020 (0.933)	−0.288 (0.217)	**0.372** (0.106) [−0.098, 0.707]

*Note:* Ns for all flexibilities is 20, except for Cognitive flexibility—Switching (*N* = 16). Polarities are reversed for both Cognitive Flexibility measures; Switching and Inhibition, with higher scores indicating greater flexibility. The correlations highlighted in green indicate positive correlations ≥ 0.30, and the correlations highlighted in red indicate negative correlations ≤ −0.30. Exact *p*‐values are in parentheses (), and confidence intervals of the correlations (≤ −0.30 or ≥ 0.30) are in square brackets []. All correlations are Pearson correlations, except the correlations with Healthcare Consumption, which are Spearman correlations.

** *p* < 0.01.

Closer examination of the correlation analyses of the different phenotypes of psychological flexibility and health indicators shows that four (cognitive flexibility‐switching, expressive flexibility, coping flexibility‐versatility, goal reengagement) of the eight flexibility measures consistently showed the expected direction in the moderate to strong associations (*r* = −0.35 to *r* = 0.60), both for positive and negative health indicators. For two flexibility measures (psychophysiological flexibility and goal disengagement), the moderate correlations (*r* = −0.47 to *r* = 0.40) consistently showed a relationship in the unexpected direction with both positive and negative health indicators; a higher level of these two flexibility measures was associated with a lower level of positive health indicator(s) and a higher level of negative health indicator(s). One flexibility measure (coping flexibility‐reflective) showed no relationship at all with health indicators and one flexibility measure (cognitive flexibility‐inhibition) showed a strong relationship (*r* = −0.58) in the expected direction and a moderate relationship (*r* = −0.42) in the non‐expected direction.

Furthermore, one flexibility measure (flexibility‐versatility) showed a moderate correlation with 83% (10/12) of the health indicators, while all other flexibilities showed a maximum of six or fewer (≤ 50%) moderate to strong correlations with health indicators. When analyzing more specific health domains (mental, physical), there were only two flexibility measures (coping flexibility‐versatility, goal reengagement) that showed moderate correlations with mental health indicators (mental well‐being, anxiety), meaning that higher levels of these flexibility measures were associated with higher mental well‐being (*r* = 0.32, *r* = 0.31) and lower levels of anxiety (*r* = −0.34, *r* = −0.31) in a group of patients with chronic pain. Regarding health indicators in the physical domain (pain, fatigue), there were also two flexibility measures (coping flexibility‐versatility, goal disengagement) that showed moderate correlations. Higher levels of coping flexibility‐versatility were moderately negatively related to pain (*r* = −0.34) and fatigue (*r* = −0.34), while goal disengagement was positively related to fatigue (*r* = 0.41). Finally, four flexibility measures (cognitive flexibility‐inhibition, coping flexibility‐versatility, expressive flexibility, goal disengagement) showed a moderate to strong positive or negative correlation (*r* = −0.58 to *r* = 0.36) with pain catastrophizing.

## 4 Discussion and Conclusion

### 4.1 Discussion

The primary aim of this study was to evaluate the feasibility of assessing psychological flexibility in all its phenotypical characteristics in a small heterogeneous sample of patients with chronic pain and in addition to explore its correlates. It was feasible to test the research protocol and it was easily applicable. No adverse events were reported. There were no complaints about the length or design, and dropout was very low. The instruments provided valid measurements of the different psychological flexibilities and positive and negative health indicators, showing sufficient variability. Overall, it can be concluded that the research design seems feasible to carry out in a larger study.

Besides feasibility, this study provides some preliminary results with indications for relevant further research. First of all, this is the first study to our knowledge that demonstrates relationships between five different phenotypes of psychological flexibility. Since the majority (79%) of the different psychological flexibility measures show no or weak (> −0.30 or < 0.30) correlations, it is suggested that the different phenotypes of psychological flexibility are indeed measuring different individual characteristics and therefore add relevant information on top of each other.

We did, however, find strong correlations (≤ −0.50 or ≥ 0.50) between the two concepts of coping flexibility (versatility and reflective coping), between coping flexibility‐versatility and goal reengagement, and between cognitive flexibility‐inhibition and psychophysiological flexibility (HRV), which are generally in line with previous literature in chronic pain or other populations [42, 55, 56]. As Vriezekolk et al. [42] have suggested, the correlation between the two subscales of the COFLEX (versatility and reflective coping) can be considered lower‐order factors of the higher construct coping flexibility. The strong correlation between coping flexibility‐versatility and goal reengagement can be explained by the fact that the COFLEX questionnaire is partly based on the principles of another goal adjustment questionnaire: the Tenacious Goal Pursuit (TEN) and Flexible Goal Adjustment (FLEX) scales [57]. The coping flexibility‐versatility scale measures the ability to flexibly use both coping strategies (TEN and FLEX) in accordance with personal goals and situational limitations. Some items of the coping flexibility‐versatility scale in the COFLEX are thus based on flexibly adjusting unattainable goals, which resembles items of the goal reengagement capacities (identify and commit to new goals and start pursuing them) of the GAS questionnaire [22], which is a possible explanation for this strong interrelationship. The correlation between cognitive flexibility‐inhibition and HRV is in line with findings in the literature [55, 56] in which various cognitive functions, including inhibition, are positively related to resting HRV. Age can possibly have a moderating effect on this correlation, where both cognitive functions and HRV decline with age [58, 59, 60].

Although no definitive conclusions can be drawn based on the small sample in this study, overall it can be concluded that including all five phenotypes of psychological flexibility in follow‐up research is relevant to provide an encompassing overview of an individual's psychological flexibility.

Besides the interrelationships between the different phenotypes of psychological flexibility, results show that 70% (21 of the 30) of the moderate to strong correlations (≤ −0.30 or ≥ 0.30) between the psychological flexibility measures and positive and negative health indicators were in the expected direction, namely higher psychological flexibility scores were related to higher positive and lower negative health indicators. These results provide a preliminary but promising finding that psychological flexibility is associated with better adaptation to chronic pain, and findings are consistent with most previous research in chronic pain populations and several other populations [27, 34, 61, 62, 63, 64, 65, 66]. This may be especially important for interventions that focus on increasing the adaptive capacity with health outcomes as a result [67, 68, 69] such as multidisciplinary rehabilitation programs, where reducing distress, optimizing chronic pain management, and increasing individual patient participation levels are central. The current study also showed that higher cognitive, expressive, and coping flexibility was associated with less pain catastrophizing, which is a very important risk factor in the development of chronic pain and is associated with different (pain‐related) outcomes [70]. As catastrophizing indicates rigid cognitive and emotional responses to pain, it can conceptually be seen as the opposite of flexibility, explaining their negative association.

Finally, if future research shows that (a combination of) different phenotypes play a role in the development or maintenance of chronic pain, for example after surgery, pre‐operative screening and treatment may contribute to reducing post‐operative chronic pain. For example, we know that cognitive flexibility is a predictor of less post‐operative pain after joint surgery [31, 71].

An unexpected finding was that in 30% (9 of the 30) of the results, higher psychological flexibility scores were moderately to strongly associated with lower scores on positive health indicators and higher scores on negative health indicators. Higher goal disengagement was correlated with less participation in work and education, more fatigue and catastrophizing about pain, and higher healthcare consumption, which is contrary to the results of the meta‐analysis of Barlow, Wrosch, and McGrath [65], but in line with other studies [72, 73, 74]. Goal adjustment may have more to do with long‐term regulation of higher‐order goals that an individual pursues (throughout life) than with adaptation to everyday situations [22]. In this way, it may conceptually differ from the other flexibility measures. A specific characteristic of the research sample and timing of the measurements in this study are possible explanations. Since these patients have experienced pain for a long time (75% more than 2 years in this sample), without much treatment result, they may realize ‐being referred to an academic pain center‐ they have to disengage former goals in life, but do not know yet what the future will look like. This may be associated with more catastrophizing, doctor visits, fatigue, and less participation. Another possible explanation is found in research by Ramírez‐Maestre et al. [72]. Their research has also shown that higher levels of goal disengagement are associated with more rumination (part of pain catastrophizing) in patients with chronic pain. They indicate that a possible cause is that patients continue to ruminate about previously abandoned (unattainable) goals. Pain catastrophizing can also play a (mediating) role in the negative relationship between goal disengagement and participation in Work and Education in this study. A meta‐analysis shows the role of pain catastrophizing in pain‐related disability [75], which is also found in the research of Ramírez‐Maestre [72].

Since this is a correlational study, no statements can be made about causality. Perhaps a higher level of fatigue or more frequent use of health care leads to a realization that different choices have to be made for a more balanced life, and this goes hand in hand with disengagement from goals, whether or not mediated by catastrophizing [76].

The negative correlation between cognitive flexibility‐inhibition and participating outdoors may be explained by the moderating effect of age as well, since cognitive functions (e.g., inhibition) decline with age. Younger people may experience more limitations in participation outdoors when they compare themselves with peers than older people. Psychophysiological flexibility (HRV) showed a negative correlation with participation (indoor, outdoor, family role) and a positive correlation with healthcare consumption. A possible explanation for this may be that a higher resting HRV reflects a higher level of physical activity of a person [77, 78]. If there is a higher level of physical activity, then more limitations may be experienced to participate due to the pain. The same could apply to healthcare consumption. When pain interferes more with daily life in physically more active people, reflected by a higher HRV, it may generate more doctor visits to stay as active as possible. Another explanation for the unexpected positive association between HRV (and the same applies to Goal disengagement) and healthcare consumption may lie in the methodological choice to measure healthcare consumption in categorical variables. Two categories (6–10 doctor's visits last 12 months, more than 20 doctor's visits last 12 months) appear to account for 40% of the responses each. The lack of variance of this variable combined with the small sample size of this study may reduce the reliability of the correlation coefficient, making consistent and reproducible conclusions difficult. It may also limit the ability to identify detailed relationships, which may complicate interpretation.

### 4.2 Limitations

Although the feasibility of the research protocol has been demonstrated, the generalizability and validity of the results of the associations between the different psychological phenotypes and their associations with different health outcomes are limited due to the cross‐sectional design and the heterogeneous small sample. Longitudinal follow‐up studies in a representative homogeneous sample reduce bias, increase reliability, and provide more insight into cause and effect. If psychological flexibility also plays a role in the development of chronic pain, it may be potentially useful to assess psychological flexibility in situations where patients are at risk for developing chronic pain (e.g., before surgery). The validity of this should also be tested in a prospective study design.

This study did not account for potential confounders (e.g., medication use, basal activity levels, comorbidities). For follow‐up studies, it is recommended to control for this to reduce possible bias and increase reliability and generalizability. Besides, stratified analyses (e.g., age, gender, number of life stressors, pain severity, pain duration) may provide more insight into differences in psychological flexibility based on demographic or clinical subgroups.

Although not all specific to this study, there are also some weaknesses in the measurement of cognitive flexibility and HRV. Twenty percent of the respondents did not meet the cut‐off score to participate in the task‐switching test, leading to invalid measurements of this part of cognitive flexibility. HRV measurements were successful in 19 of 20 participants, with the remaining participant producing a noisy, and thus less stable measurement. HRV measurements were on the short side, and we did not consider the influence of confounding factors (e.g., alcohol, caffeine) [35].

The HRV measurement was shortened due to the overall length of the protocol and, given the scope of this study, the most important thing was to get an impression of the feasibility of measuring HRV using a chest strap and a phone app. Although there is evidence that root mean square of successive differences (RMSSD) values from short HRV measurements (1 min) are as reliable as those from standard 5‐min measurements [43], this is a weakness of the current study.

Another limitation of this study is that the measurements were carried out at different times, thus causing methodological problems. Some measures (depression, anxiety, and pain catastrophizing) were taken online before the appointment at the pain center, whereas the rest of the measurements were all performed after the physician's appointment at the pain center. Besides this, a physician's visit is potentially stressful, which may interfere specifically with the neuropsychological tests [79] and HRV‐scores [80]. In follow‐up research, it is recommended to obtain more stable data for task‐switching, to take a longer‐term and thus more reliable measurement of HRV, and to perform the entire research protocol at the same time and at a preferably non‐distressing moment.

Due to the five separate scales of the measure of participation and the lack of a total score, it was difficult to unequivocally conclude whether there is a trend between the different phenotypes of psychological flexibility and participation. In follow‐up research, it is advised to use a different instrument than the IPA for interpretation matters.

Valuable patient insights were not used in the design of this study. For future studies on this topic, it would be advised to involve patients in the design of the longitudinal study to best align the research question and outcome measures with patients' needs and priorities and to receive additional suggestions for recruiting respondents.

Finally, an important aspect of many flexibility models or theories is the continuous flexible interaction between person and situational demands. In the current cross‐sectional study, environmental factors as such were not taken into account. The person's ability to be or maintain healthy also depends on the amount or severity of stressors they experience or resources a person has [81, 82]. Both stressors (nature, size, quantity) and (social) resources (support from family or friends, stable relationships, stable income, etc.) that cause or reduce stress can therefore be a good addition to follow‐up research.

### 4.3 Conclusion

The primary aim of this study, feasibility of measuring the different phenotypes of psychological flexibility with the described research protocol, has been achieved. In addition, the results of the study provide sufficient support to include all five different phenotypes of psychological flexibility in further studies. Although generalizability and validity are limited due to the small sample, the results provide preliminary indications that all phenotypes are unique constructs and that they all, more or less, play a potential role in adaptation to chronic pain, as indicated by different health outcomes.

Future longitudinal studies can provide more insight into whether and to what extent different phenotypes of psychological flexibility are actually important factors in effectively adapting to or preventing chronic pain.

### 4.4 Practice Implications

These results suggest the potential importance of psychological flexibility in optimal adaptation to chronic pain, making it a possible (psychotherapeutic) target in the treatment of patients. In line with prior research, all phenotypes of psychological flexibility have been described as potential targets in treatment to learn to adapt to chronic pain or reduce symptoms [67, 68, 69, 83, 84, 85, 86, 87, 88]. Biofeedback training or mindfulness‐based interventions may improve autonomic regulation; by performing a cognitive training, cognitive flexibility can be improved, while therapies such as cognitive behavioral therapy may promote adaptive coping strategies and emotion regulation. Finally, in the context of adaptation, it may be important to focus on letting go of unachievable goals and formulating achievable goals, especially within rehabilitation programs for patients with chronic pain. Interventional studies targeting (a combination of) psychological flexibility phenotypes should lead to measurable improvements in pain management outcomes. Finally, if psychological flexibility also plays a buffering role in the development of chronic pain, it may be potentially useful to assess psychological flexibility before surgery and provide prehabilitation training aimed at increasing it.

## Author Contributions

**Kris C. P. Vissers:** conceptualization, data curation, formal analysis, funding acquisition, investigation, methodology, supervision, writing – review and editing. **Monique A. H. Steegers:** conceptualization, formal analysis, funding acquisition, investigation, methodology, supervision, writing – review and editing. **Henriët van Middendorp:** conceptualization, formal analysis, funding acquisition, investigation, methodology, supervision, writing – review and editing. **Martijn F. Pisters:** funding acquisition, supervision, writing – review and editing. **Ewald M. Bronkhorst:** data curation, formal analysis, supervision, writing – review and editing. **Maike Borgonjen:** data curation, formal analysis, project administration. **Ivo P. Legierse:** conceptualization, data curation, formal analysis, funding acquisition, investigation, methodology, project administration, visualization, writing – original draft.

## Ethics Statement

The authors have nothing to report.

## Consent

All patients were between 18 and 70 years of age and were willing and able to participate in this study on a completely voluntary basis. Written informed consent was obtained from all patients.

## Conflicts of Interest

Prof. Dr. Vissers is an Editorial Board member of Pain Practice and a co‐author of this article. To minimize bias, he is excluded from all editorial decision‐making related to the acceptance of this article for publication.

## Data Availability

The data that support the findings of this study are available on request from the corresponding author. The data are not publicly available due to privacy or ethical restrictions.
